# In-hospital outcomes and associated factors of mortality in thai children with diabetic ketoacidosis: A national data analysis 2015–2023

**DOI:** 10.1371/journal.pone.0342777

**Published:** 2026-02-13

**Authors:** Ratikorn Chaisiwamongkol, Rattapon Uppala, Phanthila Sitthikarnkha, Leelawadee Techasatian, Suchaorn Saengnipanthkul, Pope Kosalaraksa, Kaewjai Thepsuthammarat, Sirapoom Niamsanit

**Affiliations:** 1 Department of Pediatrics, Faculty of Medicine, Khon Kaen University, Khon Kaen, Thailand; 2 Clinical Epidemiology Unit, Faculty of Medicine, Khon Kaen University, Khon Kaen, Thailand; Pelita Harapan University Faculty of Medicine: Universitas Pelita Harapan Fakultas Kedokteran, INDONESIA

## Abstract

**Background and aims:**

Diabetic ketoacidosis (DKA) remains a major cause of pediatric morbidity and death. This study examined national trends in DKA hospitalizations and factors associated with in-hospital mortality among Thai children.

**Materials and methods:**

A nationwide, retrospective cohort study was conducted using data from the National Health Security Office (NHSO) during 2015–2023. Children aged 1 month to under 18 years hospitalized with DKA were identified using International Classification of Diseases, 10th Revision, Thai Modification (ICD-10-TM) codes. Prevalence and mortality were described by year and region. Factors associated with death were assessed with multivariable logistic regression; model discrimination used area under the curve (AUC).

**Results:**

Among 10,669 admissions, national DKA prevalence increased from 4.5 to 11.8 per 10,000 pediatric hospitalizations, with Bangkok showing the highest rates. The overall intubation rate was 10.2%, peaking in infants and older adolescents. Although national mortality declined from 2.2% to 0.6%, regional and age-specific fluctuations persisted. Independent associations with mortality included malignancy (Adjusted odds ratio [AOR] 5.25, 95% CI: 1.63-16.92; p=0.005), septic shock (AOR 2.93, 95% CI: 1.71-5.03; p < 0.001), multiorgan dysfunction syndrome (MODS) (AOR 9.46, 95% CI: 5.44-16.46; p < 0.001), and need for intubation (AOR 33.43, 95% CI: 17.14-65.22; p < 0.001). Compared with type 1 diabetes, type 2 (AOR 1.64, 95% CI: 1.00-2.69) and other/unspecified diabetes (AOR 2.34, 95% CI: 1.25-4.38) had higher odds of death. Model performance was excellent (AUC 0.9639; pseudo-R² 0.5245).

**Conclusion:**

DKA hospitalizations are increasing in Thailand, with regional variation and persistent mortality risk, particularly among patients with critical complications and vulnerable groups. Although declining mortality trends and lower mortality in recurrent cases suggests improved protocol-based treatment, targeted prevention strategies remain essential for high-risk populations.

## Introduction

DKA is a potentially life‑threatening acute metabolic emergency in children and adolescents. Despite established, evidence-based management pathways—most notably the International Society for Pediatric and Adolescent Diabetes (ISPAD) guidelines—DKA remains the principal cause of diabetes-related mortality in youth, with cerebral edema as the most devastating complication [1–3]. In high-resource settings, in-hospital mortality has fallen to <1%, yet severe presentations and delayed recognition still drive substantial morbidity and mortality worldwide [4–5].

In Thailand, recent registry and population-based reports indicate high severity at diagnosis and rising DKA admissions among youth, but contemporary, nationally representative analyses of mortality trends and clinical predictors are limited [6–8]. Given universal health coverage and heterogeneous resource distribution across the country, up-to-date national data are essential to target system-level improvements [9–11].

To address these gaps, we conducted a nationwide, retrospective analysis of pediatric DKA hospitalizations in Thailand (2015–2023) using the NHSO database. Our objectives were to (1) describe national trends in DKA admissions and in-hospital mortality and (2) identify clinical factors—such as cerebral edema, shock, and organ dysfunction—associated with in-hospital death, to inform policy and guide improvements in care for Thai children with DKA.

## Materials and methods

### Study design and participants

This nationwide retrospective study analyzed data from the NHSO database, covering pediatric hospitalizations across Thailand from January 1, 2015, to December 31, 2023. The NHSO manages the Universal Coverage (UC) scheme, which provides health services for approximately 72% of Thailand’s population [9].

The Thai healthcare system comprises public and private hospitals organized into three levels. Primary care hospitals deliver basic outpatient services and serve as the first point of contact. Secondary hospitals are equipped for more specialized care, often with pediatric wards, but typically lack pediatric endocrinologists and intensive care specialists. Tertiary hospitals act as referral centers and are equipped with pediatric intensive care units (PICUs) and full subspecialist support. Private hospitals vary in capacity; some operate at a tertiary level, while others provide services comparable to secondary facilities.

Eligible patients included children aged between 1 month and less than 18 years who were hospitalized with a diagnosis of DKA. DKA cases were identified using the ICD-10-TM codes: E101 (insulin-dependent diabetes mellitus with ketoacidosis), E111 (non-insulin-dependent diabetes mellitus with ketoacidosis), E131 (other specified diabetes mellitus with DKA), and E141 (unspecified diabetes mellitus with DKA). Diagnoses were assigned by attending physicians. Although some variation in clinical coding practices may exist, the NHSO conducts routine audits of submitted claims to verify diagnostic accuracy and minimize misclassification. This study adhered to the Strengthening the Reporting of Observational Studies in Epidemiology (STROBE) guidelines [12].

### Demographic data collection

Demographic and clinical variables were extracted from the NHSO database. Collected variables included age, sex, year of admission, hospital level, and geographical regions; records with incomplete demographic identifiers were excluded. Recurrent admissions were defined as multiple hospitalizations of the same patient during the study period; however, linkage to admissions occurring before the study window was not feasible. The underlying type of diabetes mellitus associated with DKA was categorized according to ICD-10-TM codes: type 1 diabetes mellitus (E101), type 2 diabetes mellitus (E111), and other or unspecified types (E131 and E141).

Comorbidities, DKA complications, and organ dysfunctions were identified from ICD-10-TM codes, including cerebral edema (G93.6), cardiovascular dysfunction/shock (R57), acute renal failure (N17–N17.9), and acute liver failure (K72.0–K72.9). MODS was defined as dysfunction involving more than one organ system. Critical care interventions were identified using the International Classification of Diseases, 9th Revision, Clinical Modification (ICD-9-CM) procedure codes, including endotracheal intubation (96.04, 96.71, 96.72) and renal replacement therapy (hemodialysis: 39.95; peritoneal dialysis: 54.98). In this dataset, the absence of a specific diagnosis or procedure code was interpreted as the condition or intervention not occurring during that admission.

### Statistical analyses

All statistical analyses were conducted using Stata version 18 (Stata Corp LLC, College Station, TX, US). Categorical variables were presented as frequencies and percentages, while continuous variables were summarized using means and standard deviations (SD) or medians and interquartile ranges (IQR), based on distribution determined by the Shapiro–Wilk normality test.

The annual prevalence of DKA hospitalization was calculated by dividing the number of DKA admissions by the total number of pediatric hospitalizations each year, expressed per 10,000 admissions. The in-hospital mortality rate was determined by dividing the number of DKA-related deaths by the total number of DKA admissions in the corresponding year and expressed as a percentage.

Patients were categorized into five age groups (1 month to <1 year, 1 to <5 years, 5 to <10 years, 10 to <15 years, and 15 to <18 years) for age-stratified mortality analysis. Univariate logistic regression was initially employed to identify potential predictors of in-hospital mortality. Variables with p < 0.20 entered the multivariable logistic regression, and backward elimination was applied with retention at p < 0.10. Multicollinearity was assessed using variance inflation factors (VIF); predictors with acceptable collinearity were defined as VIF ≤ 10. COR, AOR, and 95% confidence intervals were reported. A two-sided p-value of less than 0.05 was considered statistically significant. Model performance was assessed by discrimination (AUC) and calibration (Hosmer–Lemeshow test). To test robustness, exploratory interaction terms among clinically plausible predictors were evaluated in sensitivity analyses, and full results are provided in the S1 and S2 tables in S1 File.

### Ethics approval

The study was approved by the Human Research Ethics Committee of Khon Kaen University (approval number: HE681330). Data were accessed for research purposes on 7 August 2025. Since all data were de-identified and obtained from a secondary administrative database, the requirement for informed consent was waived.

## Results

### Demographic and clinical characteristics

**Fig 1:**
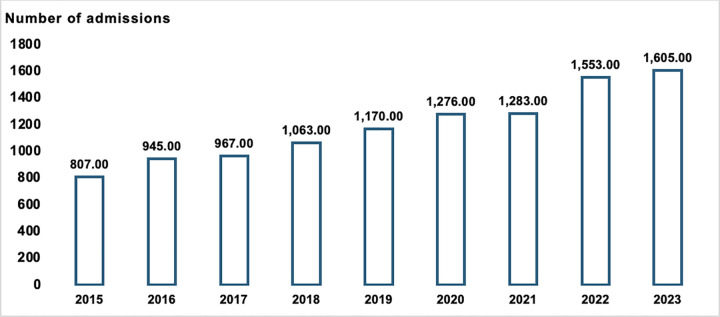
Number of admissions per year of Thai children diagnosed with DKA from 2015 to 2023.

A total of 10,669 hospital admissions for DKA among Thai children aged less than 18 years were identified during the nine-year study period from 2015 to 2023. The annual number of DKA admissions showed a consistent upward trend throughout the study duration. Specifically, there were 807 admissions in 2015, which steadily rose to 1,605 cases by 2023. The number of cases surpassed 1,000 for the first time in 2018 and continued to increase annually thereafter, with the largest spike observed in 2022 and 2023 (**Fig 1**).

**Table 1** summarizes the demographic and baseline clinical characteristics of children hospitalized with DKA during the study period. Of the total admissions, 38.4% were male. Most patients were adolescents, with 46.9% aged 10–14 years and 32.7% aged 15–17 years. Only 0.4% were infants younger than one year of age. The northeast region accounted for the highest proportion of admissions (33.3%), followed by the central region (29.5%), the southern region (14.6%), the northern region (12.3%), and Bangkok (10.4%).

**Table 1:** Demographic data of hospitalized Thai children due to DKA from 2015–2023.

Factors	n = 10,669 (%)
**Male**	4,094 (38.4)
**Age group**	
1) 1 month to <1 year	42 (0.4)
2) 1 to <5 years	541 (5.1)
3) 5 to <10 years	1,595 (15.0)
4) 10 to <15 years	5,001 (46.9)
5) 15 to <18 years	3,490 (32.7)
**Region**	
1) Bangkok	1,105 (10.4)
2) Central	3,146 (29.5)
3) Northeast	3,552 (33.3)
4) North	1,311 (12.3)
5) South	1,555 (14.6)
**Hospital level**	
1) Primary	703 (6.6)
2) Secondary level	4,671 (43.8)
3) Tertiary	5,086 (47.7)
4) Private	209 (2.0)
**Underlying types of diabetes mellitus**	
1) Type 1 diabetes mellitus	8,314 (77.9)
2) Type 2 diabetes mellitus	1,744 (16.4)
3) Other or unspecified types	614 (5.8)
**Co-morbidities and co-diagnosis**	
1) Chronic Respiratory Disease	77 (0.7)
2) Congenital heart disease	16 (0.2)
3) Malignancy	62 (0.6)
4) Malnutrition	86 (0.8)
5) Septic shock	176 (1.6)
**Obesity**	
1) Simple obesity	170 (1.6)
2) Morbid obesity	52 (0.5)
**Recurrent admissions**	7,560 (70.9)
**Complications and organ dysfunctions**	
1) Cerebral edema	59 (0.6)
2) Hypokalemia	3,718 (34.9)
3) Cardiovascular dysfunction	220 (2.1)
4) Acute renal failure	284 (2.7)
5) Acute liver failure	10 (0.1)
6) MODS	435 (4.1)
**Need intubation**	1,088 (10.2)
**Need renal replacement therapy**	31 (0.3)
**Death**	138 (1.3)
**Hospital Length of Stay (days), median (IQR)**	5 (3, 8)

Regarding healthcare service levels, nearly half of all cases (47.7%) were admitted to tertiary hospitals, and 43.8% to secondary hospitals. The majority of patients were diagnosed with type 1 diabetes mellitus (77.9%), while 16.4% were diagnosed with type 2 diabetes mellitus and 5.8% with other or unspecified types. Recurrent admissions were common, with 70.9% of patients experiencing at least one readmission for DKA during the study period. A small proportion of patients had pre-existing comorbidities, including chronic respiratory disease (0.7%), congenital heart disease (0.2%), malignancy (0.6%), and malnutrition (0.8%). Obesity was reported in 2.1% of cases, with 1.6% having simple obesity and 0.5% classified as morbidly obese.

Several DKA-related complications and associated organ dysfunctions were observed with hypokalemia being the most frequently recorded (34.9%). Other notable complications included cardiovascular dysfunction in 2.1%, acute renal failure in 2.7%, and cerebral edema in 0.6%. MODS was present in 4.1% of cases. Critical care interventions were required in a substantial proportion of patients. A total of 1,088 children (10.2%) underwent endotracheal intubation, while 31 patients (0.3%) received renal replacement therapy. Overall, 138 in-hospital deaths were recorded, corresponding to a case fatality rate of 1.3%.

**Fig 2:**
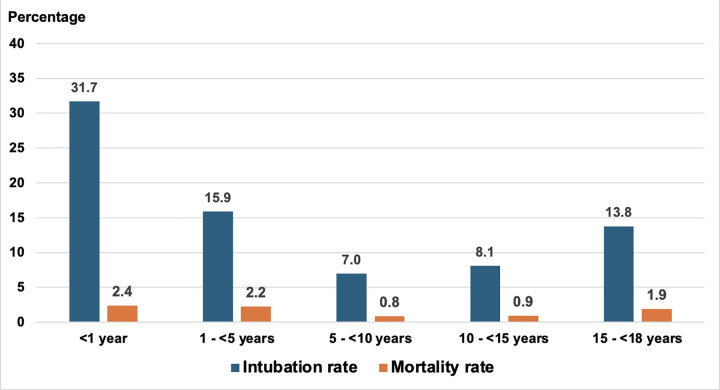
Mortality and intubation rates of pediatric DKA, stratified by age group.

Further analysis of mortality and intubation rates stratified by age group is shown in **Fig 2**. The highest mortality rates were observed among children under five and older adolescents aged 15–17 years. Similarly, the need for endotracheal intubation was more common in these age groups.

### Geographic variation in DKA prevalence and mortality across Thai regions

**Fig 3:**
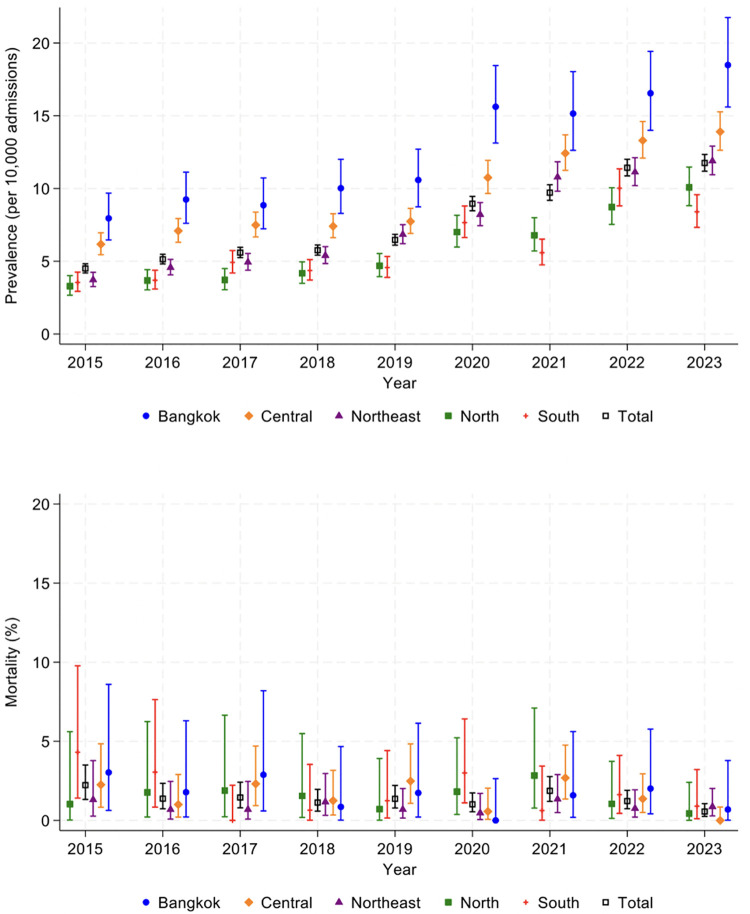
Trends in the prevalence and mortality of pediatric DKA in Thailand from 2015 to 2023, stratified by geographic region. *The 95% CIs, depicted as bars in the figure.

Between 2015 and 2023, the national prevalence of pediatric DKA admissions in Thailand demonstrated a substantial and consistent increase, rising from 4.5 to 11.8 cases per 10,000 pediatric hospitalizations **(Fig 3**). This upward trend was observed across all geographic regions, though the magnitude varied. Bangkok persistently reported the highest prevalence throughout the study period, nearly doubling from 8.0 per 10,000 admissions in 2015 to 18.5 in 2023. The Central and Northeast regions followed similar trajectories, increasing from 6.2 to 13.9 and 3.7 to 11.9 per 10,000 admissions, respectively. The North and South, which had the lowest initial prevalence in 2015 (3.3 and 3.5, respectively), also showed gradual but notable increases, reaching 10.1 and 8.4 per 10,000 admissions by 2023.

Despite the rising number of admissions, the overall national in-hospital mortality rate from pediatric DKA declined, from 2.2% in 2015 to 0.6% in 2023. However, region-specific mortality trends exhibited considerable fluctuations throughout the study period, with no single region consistently demonstrating the lowest or highest mortality. The South region displayed marked variation, recording the highest mortality in 2015 (4.3%) and again in 2020 (3.0%), but also near-zero mortality in 2017. Bangkok similarly experienced variable outcomes, peaking at 3.0% in 2015 and 2.9% in 2017, before declining to 0.7% by 2023. The Central region showed relatively low mortality in the early years (1.0% in 2016) and reported near-zero deaths in 2023 but reached 2.7% in 2021. The Northeast, which contributed the largest number of DKA admissions, generally maintained a lower and more stable mortality rate, ranging from 0.7% to 1.3% over the nine years. The North also demonstrated relatively low mortality overall, though it experienced a temporary peak of 2.8% in 2021.

### Factors associated with in-hospital mortality

**Table 2** presents the univariate and multivariate analyses of factors associated with in-hospital mortality. In the univariate analysis, several features showed associations with death: male sex (COR 1.48, 95% CI: 1.06–2.07; p = 0.022); diabetes type-compared with type 1, type 2 (COR 2.07, 95% CI: 1.38–3.11; p < 0.001) and other/unspecified (COR 4.42; 95% CI: 2.79–7.01; p < 0.001) had higher crude odds of death; malignancy (COR 13.79, 95% CI: 6.66–28.55; p < 0.001); septic shock (COR 118.34, 95% CI: 80.25–174.52; p < 0.001); cerebral edema (COR 31.99; 95% CI: 17.54–58.35; p < 0.001); cardiovascular dysfunction (COR 106.24, 95% CI: 72.81–155.03; p < 0.001); acute renal failure (COR 34.21, 95% CI: 23.81–49.14; p < 0.001); MODS (COR 90.40, 95% CI: 60.68–134.70; p < 0.001); and need for intubation (COR 114.97, 95% CI: 61.88–213.64; p < 0.001).

**Table 2:** Factors associated with mortality among children hospitalized with DKA in Thailand from 2015–2023.

Factors	COR	95% CI	p-value	AOR	95% CI	p-value
**Male**	1.48	1.06, 2.07	0.022	1.26	0.83, 1.91	0.288
**Age group**			<0.001	Multicollinearity		
1) 1 month to <1 year	1					
2) 1 to <5 years	0.93	0.12, 7.33				
3) 5 to <10 years	0.34	0.04, 2.64				
4) 10 to <15 years	0.38	0.05, 2.83				
5) 15 to <18 years	0.79	0.11, 5.83				
**Underlying types of diabetes mellitus**			<0.001			0.010
1) Type 1 diabetes mellitus	1			1		
2) Type 2 diabetes mellitus	2.07	1.38, 3.11		1.64	1.00, 2.69	
3) Other or unspecified types	4.42	2.79, 7.01		2.34	1.25, 4.38	
**Comorbidities and co-diagnosis**						
1) Chronic Respiratory Disorder	1.00	0.14, 7.27	0.997			
2) Congenital heart disease	5.12	0.67, 39.01	0.079			
3) Malignancy	13.79	6.66, 28.55	<0.001	5.25	1.63, 16.92	0.005
4) Malnutrition	NA		0.286			
5) Septic shock	118.34	80.25, 174.52	<0.001	2.93	1.71, 5.03	<0.001
**Complications and organ dysfunctions**						
1) Cerebral edema	31.99	17.54, 58.35	<0.001	1.99	0.91, 4.35	0.085
2) Cardiovascular dysfunction	106.24	72.81, 155.03	<0.001			
3) Acute renal failure	34.21	23.81, 49.14	<0.001			
4) MODS	90.40	60.68, 134.70	<0.001	9.46	5.44, 16.46	<0.001
**Need intubation**	114.97	61.88, 213.64	<0.001	33.43	17.14, 65.22	<0.001

**Pseudo R**^**2**^ **= 0.5245, AUC =** 0.9639.

In the multivariate model, the strongest association was intubation (AOR 33.43, 95% CI: 17.14–65.22; p < 0.001), followed by MODS (AOR 9.46, 95% CI:5.44–16.46; p < 0.001) and malignancy (AOR 5.25, 95% CI:1.63–16.92; p = 0.005). Diabetes type also remained important compared with type 1 diabetes (type 2: AOR 1.64, 95% CI: 1.00–2.69; other/unspecified: AOR 2.34, 95%CI: 1.25–4.38). Septic shock was independently associated with mortality (AOR 2.93, 95% CI: 1.71–5.03; p < 0.001). Cerebral edema did not meet significance after adjustment (AOR 1.99, 95% CI: 0.91–4.35; p = 0.085).

Multicollinearity was assessed using variance inflation factors: most covariates were acceptable (VIFs ~ 1–7; mean 8.91), while age group indicators exceeded the conventional threshold (VIF > 10) and were not interpreted (S1 table in S1 File). Key predictors were stable and model discrimination was excellent (AUC = 0.96). Sensitivity models adding interaction terms between diabetes type and markers of critical illness did not show significant interactions, and discrimination was unchanged, supporting robustness of the main associations (S2 Table in S1 File).

## Discussion

In this national cohort, pediatric DKA admissions nearly doubled between 2015 and 2023, while in-hospital mortality declined from 2.2% to 0.6%. This trend is consistent with high-income settings that saw rising utilization with declining fatality during the 2000s–2010s, likely reflecting wider uptake of standardized care and critical-care capacity [13–14]. Even so, overall case fatality of 1.3% in our study sits higher than the < 0.5–1.0% often reported in high‑resource cohorts, highlighting that system‑level variation and case‑mix still matter [13].

Our finding that DKA prevalence climbed across all Thai regions from 2015 to 2023 aligns with international reports of rising pediatric DKA utilization before and during COVID‑19. The post-pandemic increase in admissions aligns with reports of higher DKA burden during 2020–2021, plausibly related to diagnostic delays and service disruption [5,15–17]. Bangkok showed the highest prevalence, but regional mortality fluctuated year-to-year without a consistently best or worst area. To steady outcomes, system actions are needed: faster referral and transport, dependable PICU readiness, and DKA-specific quality checks (timely labs, early PICU review, prompt dextrose when glucose falls) [10–11].

Age-specific risks were U-shaped: highest in children <5 years and adolescents 15–17 years, a pattern attributed to late and severe presentation or misdiagnosed with conditions like pneumonia in younger children and adherence or psychosocial factors in teens [18–22]. These findings support age-tailored prevention and escalation pathways.

In the multivariable model, endotracheal intubation, MODS, malignancy, and septic shock were each associated with higher mortality. All of these features can reflect greater DKA severity and serve as markers of extreme physiological decompensation [5,23,24]. Acute renal failure and cerebral edema, although strongly associated in univariate analysis, did not remain statistically significant after adjustment, suggesting that their mortality signal is largely captured by MODS. Diabetes types other than type 1 (type 2 and other/unspecified) were also linked to higher mortality, plausibly reflecting heterogeneous pathophysiology, co-existing comorbidities, and greater organ dysfunction [24–26]. The intubation rate in this cohort (10.2%) exceeded many high-income reports (≈1–2%), and the strong association between intubation and death is most plausibly a severity marker rather than an independent causal factor [27]. Contemporary guidance and trial data emphasize that timely recognition and careful physiologic targets (e.g., gradual osmolality correction; avoidance of severe hypocapnia) remain central to safe DKA management [3,28].

This study highlights national variation in pediatric DKA prevalence and mortality and identifies high-risk clinical features, although important limitations remain. Firstly, this dataset lacks bedside severity (pH, bicarbonate, osmolality), treatment intensity, and protocol adherence; proxy measures (e.g., intubation) may reflect airway protection or coding variation. Secondly, misclassification and under-ascertainment of complications may persist despite routine audits and would generally bias associations toward the null. Thirdly, new-onset versus established diabetes and pre-hospital deaths could not be distinguished, and unmeasured contextual factors (socioeconomic status, time-to-care, transport, PICU capacity) limit causal inference and regional comparisons. Therefore, future research should: (a) link NHSO claims with laboratory data to build a severity-adjusted DKA registry; (b) implement and evaluate ISPAD-aligned electrolyte bundles and age-specific escalation triggers; and (c) use geospatial analyses of transport and PICU access to guide regional centralization.

## Conclusions

Pediatric DKA admissions rose nationally (2015–2023), while in‑hospital mortality declined to <1% by 2023. Mortality was strongly associated with endotracheal intubation, MODS, malignancy, non-type 1 diabetes, and septic shock. Strengthening ISPAD-aligned care, rapid PICU escalation/transfer, and adolescent relapse-prevention could further reduce deaths and narrow regional variation.

## Supporting information

S1 File Variance inflation factors (VIF) estimate for covariates considered in mortality analyses. (ZIP)

## Abbreviations

AOR: Adjusted Odds Ratio
AUC: Area Under the Curve
CI: Confidence Interval
COR: Crude Odds Ratio
DKA: Diabetic Ketoacidosis
ICD‑9‑CM: International Classification of Diseases, 9th Revision, Clinical Modification
ICD‑10‑TM: International Classification of Diseases, 10th Revision, Thai Modification
IQR: Interquartile Range
ISPAD: International Society for Pediatric and Adolescent Diabetes
MODS: Multiorgan Dysfunction Syndrome
NHSO: National Health Security Office (Thailand)
PICU: Pediatric Intensive Care Unit
SD: Standard Deviation
STROBE: Strengthening the Reporting of Observational Studies in Epidemiology
UC: Universal Coverage (Thailand’s public insurance scheme)
VIF: Variance Inflation Factor
