# Supplementary material for: In-hospital outcomes and associated factors of mortality in thai children with diabetic ketoacidosis: A national data analysis 2015–2023
Source: PLoS One. 2026 Feb 13;21(2):e0342777. doi: 10.1371/journal.pone.0342777 (PMC12904397; doi:10.1371/journal.pone.0342777)
Supplement: S1 File — (ZIP) [file pone.0342777.s001.zip › S1_Table.docx]

**S1 Table.** **Variance inflation factors (VIF) estimate for covariates considered in mortality analyses**

| **Factors** | **VIF** |
| --- | --- |
| **Male** | 1.02 |
| **Age group** |  |
| 1) 1 month to <1 year | 13.33 |
| 2) 1 to <5 years | 33.59 |
| 3) 5 to <10 years | 64.63 |
| 4) 10 to -<15 years | 57.30 |
| **Underlying types of diabetes mellitus** |  |
| 1) Type 2 diabetes mellitus | 1.05 |
| 2) Other or unspecified types | 1.04 |
| **Co-morbidities and co-diagnosis** |  |
| 1) Congenital heart disease | 1.01 |
| 2) Malignancy | 1.01 |
| 3) Septic shock | 4.60 |
| **Complications and organ dysfunctions** |  |
| 1) Cerebral edema | 1.08 |
| 2) Cardiovascular dysfunction | 6.80 |
| 3) Acute renal failure | 4.17 |
| 4) MODS | 7.18 |
| **Need Intubation** | 1.19 |
