# Supplementary material for: In-hospital outcomes and associated factors of mortality in thai children with diabetic ketoacidosis: A national data analysis 2015–2023
Source: PLoS One. 2026 Feb 13;21(2):e0342777. doi: 10.1371/journal.pone.0342777 (PMC12904397; doi:10.1371/journal.pone.0342777)
Supplement: S1 File — (ZIP) [file pone.0342777.s001.zip › S2_Table.docx]

**S2 Table. Interaction between diabetes type and shock: univariate and multivariable logistic regression for in-hospital mortality among children with DKA, Thailand 2015–2023**

| **Factors** | **COR** | **95%CI** | **P-value** | **AOR** | **95%CI** | **p-value** |
| --- | --- | --- | --- | --- | --- | --- |
| **Male** | 1.48 | 1.06, 2.07 | 0.022 | 1.31 | 0.86, 2.00 | 0.208 |
| **Age group** |  |  | <0.001 | Multicollinearity | | |
| 1) 1month to <1 year | 1 |  |  |  |  |  |
| 2) 1 to <5 years | 0.93 | 0.12, 7.33 |  |  |  |  |
| 3) 5 to <10 years | 0.34 | 0.04, 2.64 |  |  |  |  |
| 4) 10 to <15 years | 0.38 | 0.05, 2.83 |  |  |  |  |
| 5) 15 to <18 years | 0.79 | 0.11, 5.83 |  |  |  |  |
| **Underlying types of diabetes mellitus (DM)** |  |  | <0.001 |  |  | 0.003 |
| 1) Type 1 DM | 1 |  |  |  |  |  |
| 2) Type 2 DM | 2.07 | 1.38, 3.11 |  | 2.48 | 1.33, 4.61 | 2.07 |
| 3) Other or unspecified types | 4.42 | 2.79, 7.01 |  | 3.04 | 1.30, 7.10 | 4.42 |
| Interaction DM*septic shock |  |  |  |  |  | 0.125 |
| Type 2 DM*shock |  |  |  | 0.35 | 0.13, 0.98 |  |
| Other Types*shock |  |  |  | 0.59 | 0.17, 2.04 |  |
| **Co-morbidities and co-diagnosis** |  |  |  |  |  |  |
| 1) Chronic Respiratory Disorder | 1.00 | 0.14, 7.27 | 0.997 |  |  |  |
| 2) Congenital heart disease | 5.12 | 0.67, 39.01 | 0.079 | 0.62 | 0.05, 7.51 | 0.71 |
| 3) Malignancy | 13.79 | 6.66, 28.55 | <0.001 | 5.50 | 1.68, 18.06 | 0.005 |
| 4) Septic shock | 118.34 | 80.25, 174.52 | <0.001 | 2.04 | 0.80, 5.19 | 0.134 |
| **Complications and organ dysfunctions** |  |  |  |  |  |  |
| 1) Cerebral edema | 31.99 | 17.54, 58.35 | <0.001 | 1.90 | 0.85, 4.24 | 0.116 |
| 2) Cardiovascular dysfunction | 106.24 | 72.81, 155.03 | <0.001 | 2.25 | 0.75, 6.70 | 0.147 |
| 3) Acute renal failure | 34.21 | 23.81, 49.14 | <0.001 | 1.08 | 0.59, 1.98 | 0.792 |
| 4) MODS | 90.40 | 60.68, 134.70 | <0.001 | 8.22 | 3.66, 18.45 | <0.001 |
| **Need intubation** | 114.97 | 61.88, 213.64 | <0.001 | 32.81 | 16.78, 64.15 | <0.001 |

**Pseudo R^2^  = 0.5291, AUC = 0.9656**
